# Developmental endothelial locus-1 in cardiovascular and metabolic diseases: A promising biomarker and therapeutic target

**DOI:** 10.3389/fimmu.2022.1053175

**Published:** 2022-11-28

**Authors:** Mengmeng Zhao, Zihui Zheng, Chenfei Li, Jun Wan, Menglong Wang

**Affiliations:** ^1^ Department of Cardiology, Renmin Hospital of Wuhan University, Wuhan, China; ^2^ Cardiovascular Research Institute, Wuhan University, Wuhan, China; ^3^ Hubei Key Laboratory of Cardiology, Wuhan, China

**Keywords:** DEL-1, cardiovascular diseases, metabolic diseases, inflammation resolution, anti-inflammation

## Abstract

Cardiovascular and metabolic diseases (CVMDs) are a leading cause of death worldwide and impose a major socioeconomic burden on individuals and healthcare systems, underscoring the urgent need to develop new drug therapies. Developmental endothelial locus-1 (DEL-1) is a secreted multifunctional domain protein that can bind to integrins and play an important role in the occurrence and development of various diseases. Recently, DEL-1 has attracted increased interest for its pharmacological role in the treatment and/or management of CVMDs. In this review, we present the current knowledge on the predictive and therapeutic role of DEL-1 in a variety of CVMDs, such as atherosclerosis, hypertension, cardiac remodeling, ischemic heart disease, obesity, and insulin resistance. Collectively, DEL-1 is a promising biomarker and therapeutic target for CVMDs.

## Introduction

A wide range of diseases that affect the heart and blood vessels are collectively referred to as cardiovascular diseases (CVDs), including atherosclerosis (AS), myocardial infarction (MI), hypertension, cardiac hypertrophy, and heart failure. Metabolic diseases, including diabetes, obesity and nonalcoholic fatty liver disease, are closely related to the occurrence and development of CVDs (1, 2). Cardiovascular and metabolic diseases (CVMDs) are the leading causes of death worldwide and result in a major socioeconomic burden on individuals and healthcare systems (3–6). These diseases are caused by a combination of multiple pathological factors, and their pathogenesis has not been fully elucidated. Although effective primary prevention and treatment strategies have reduced morbidity and mortality from CVMDs over the past 20 years, the prognosis of CVMDs remains unsatisfactory, and effective interventions are still lacking (7, 8).

Immune cells and inflammatory responses are involved in all stages of the occurrence and development of multiple CVMDs (9–11). The expression levels of various inflammatory mediators correlate with the clinical diagnosis and prognosis of CVMDs (12–18). Inflammation-related molecules such as interleukin-6 and growth differentiation factor 15 have been identified as biomarkers of CVDs (19). Regulation of immune function and the inflammatory response is an important strategy for the treatment of CVMDs (20–24). Increasing evidence shows that tissue-resident immune cells are involved in regulating the pathophysiological processes of CVMDs (25–28). Local tissues, such as vascular endothelium and adipose tissue, also have an important impact on the occurrence and development of CVMDs (29–32). Various local tissues in the human body are not only passive targets of immune and inflammatory responses but also active regulators of immunity (33). Local tissue signaling can regulate immune cell accumulation and functional plasticity and play a key role in immune-driven CVMDs (34, 35). Stromal and parenchymal cell-derived signals (including growth factors, cytokines, and other locally acting homeostatic factors) as well as intercellular adhesion interactions mediate local tissue-to-immune communication in CVMDs such as myocardial infarction (36–38). The compartmentalized expression of tissue signaling can facilitate optimal performance of cell-type-specific effects and spatial regulation of immune responses. Therefore, homeostatic molecules in the tissue microenvironment at different locations may be critical for CVMDs.

Developmental endothelial locus-1 (DEL-1) is a secreted multifunctional domain protein. As a local tissue signal, it exerts different regulatory functions in different expression regions (39). Endothelial cell-derived DEL-1 mainly regulates inflammation initiation by inhibiting neutrophil recruitment, while macrophage-derived DEL-1 promotes the resolution of inflammation by enhancing neutrophil apoptosis and macrophage efferocytosis (40). Increasing evidence has shown that the regulation of immune system homeostasis by DEL-1 plays an important role in CVMDs (41–43). In this article, we review the regulatory role of local tissue DEL-1 signaling in CVMDs and look forward to the future development of DEL-1 (**Table 1**).

**Table 1:** Roles of DEL-1 in cardiovascular and metabolic diseases.

CVMD	Animal	Animal model	Treatment	Finding	PMID
Atherosclerosis	mouse	Paigen diet for 20 weeks from the age of 24 weeks	Overall DEL-1 overexpression	Significantly reduced lipid accumulation in the aortic root; attenuated atherosclerosis	27784857
Atherosclerosis	mouse	1.Partial ligation of the left carotid artery with high fat diet for 6 weeks; 2. ApoE^−/−^ mice with high fat diet for 4 or 12 weeks	Endothelial-specific overexpression of DEL-1	Endothelial DEL-1 does not protect against atherogenesis.	28796274
Hypertension	mouse	ANGII- and DOCA-salt-induced hypertension	1. Endothelial-specific overexpression of DEL-1; 2. Recombinant DEL-1	Inhibited the progression of hypertension; Attenuated hypertension-induced cardiac remodeling	35133978
Cardiac ischemia	pig	Left circumflex artery occlusions	DEL-1 overexpression	Improved cardiac function	14530019
Myocardial infarction	mouse	Permanent ligation of the left anterior descending coronary artery	DEL-1 knock out	Ameliorated adverse cardiac healing via neutrophil extracellular traps-mediated pro-inflammatory macrophage polarization.	34375400
Ischemic hindlimb	mouse	Femoral artery excision	Recombinant DEL-1 treatment	Enhanced angiogenesis in the murine ischemic hindlimb.	14981004
Ischemic stroke	mouse	Intraluminal middle cerebral artery blockade	DEL-1 overexpression	Promoted endogenous endothelial cell proliferation and angiogenesis	18534562
Insulin resistance	mouse	High fat diet for 26 weeks	Recombinant DEL-1 treatment	Attenuated HFD-induced skeletal muscle insulin resistance.	31778646

## Expression, structure and functions of DEL-1

### Expression

DEL-1 is a 52 KD multifunctional matrix protein encoded by EDIL3 (epidermal growth factor (EGF)-like repeats and discoidin domains 3), which was cloned and characterized in angioplasty cells and early endothelial cells as early as 1998 (44). Increasing evidence shows that DEL-1 is expressed in tissues such as the brain, lung, and gums (39, 45, 46). Some tissue-resident cells, such as mesenchymal stromal cells, macrophages, neuronal cells, osteoclasts and some hematopoietic microenvironment cells, can also secrete DEL-1 (39, 40, 47, 48).

**Figure 1:**
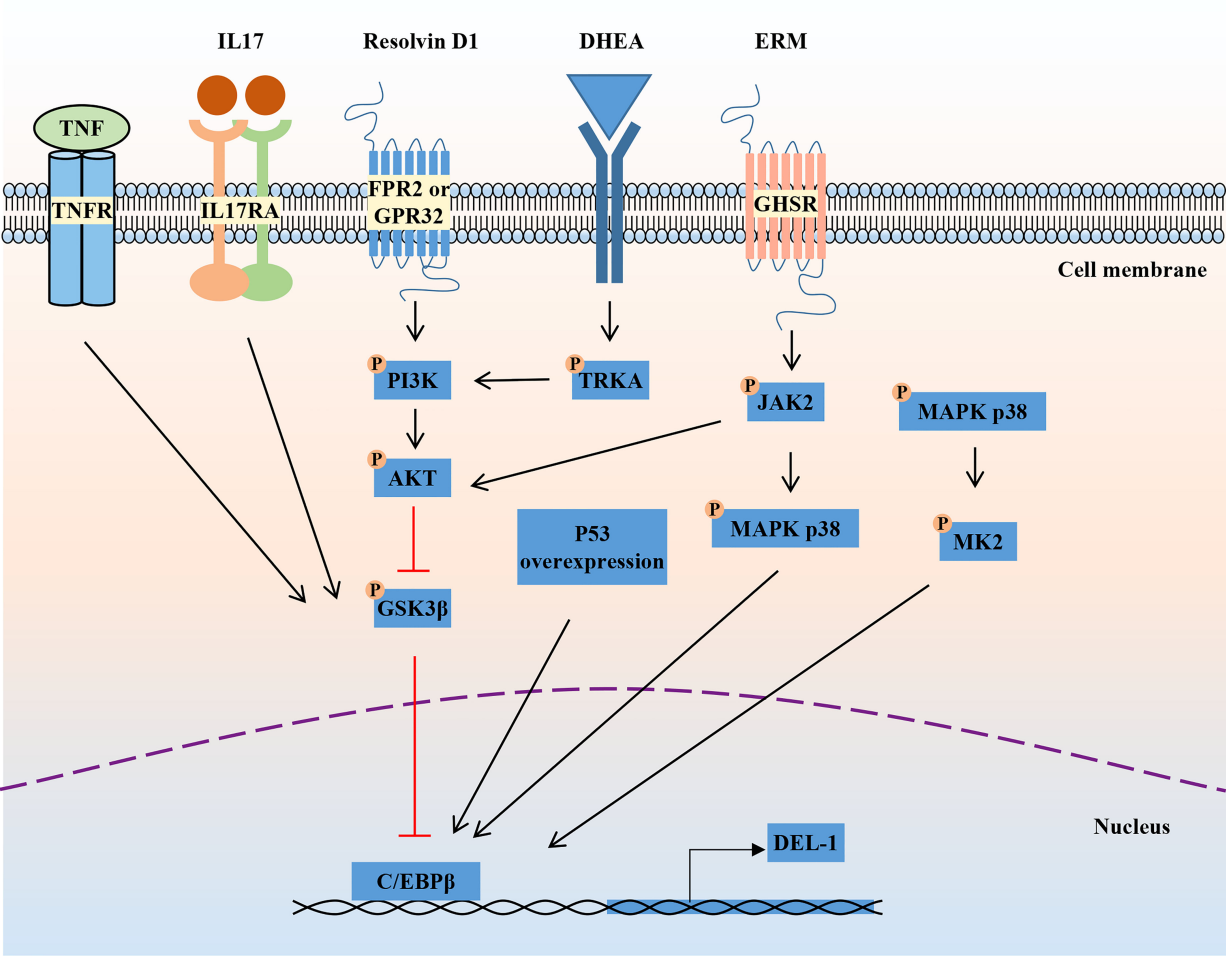
Regulation of DEL-1 expression. IL-17 and TNF reduce DEL-1 expression in a GSK3β-dependent process that inhibits the binding of the key transcription factor C/EBPβ to the EDIL3 promoter, thereby downregulating EDIL3 transcription. This inhibitory action of IL-17 can be reversed at the GSK-3β level by PI3K/AKT signaling induced by D-resolvins. Through interaction with GHSR, ERM activates JAK2 signaling, leading to DEL-1 transcription, which is MAPK p38-mediated and C/EBPβ dependent, as well as to PI3K/AKT-mediated reversal of the GSK3β-dependent inhibitory effect of IL-17 on DEL-1 expression. DHEA reduced DEL-1 expression and secretion in endothelial cells by activating TRKA and downstream PI3K/AKT signaling to restore C/EBPβ binding to the DEL-1 promoter. In addition, P53 overexpression and the activation of P38/MK2 signaling were reported to promote DEL-1 expression. DEL-1, developmental endothelial locus-1; GSK-3β, glycogen synthase kinase 3β; C/EBPβ, CCAAT/enhancer-binding protein β; PI3K, phosphoinositide 3-kinase; MAPK, mitogen-activated protein kinases; GHSR, growth hormone secretagogue receptor; ERM, erythromycin; JAK2, janus kinase 2; DHEA, dehydroepiandrosterone; TRKA, tropomyosin receptor kinase A.

The mechanism regulating DEL-1 expression in tissues has not been elucidated. The reciprocal regulatory role of IL-17 and DEL-1 is now widely recognized (**Figure 1**). IL-17 directly inhibits endothelial DEL-1 expression, thereby promoting lymphocyte function-associated antigen 1 (LFA-1)-dependent neutrophil recruitment, while DEL-1 counteracts IL-17 production and IL-17-dependent inflammation (45, 49). Mechanistically, IL-17 reduces DEL-1 expression in a glycogen synthase kinase 3β (GSK3β)-dependent process that inhibits the binding of the key transcription factor CCAAT/enhancer-binding protein β (C/EBPβ) to the EDIL3 promoter, thereby downregulating EDIL3 transcription. This inhibitory action of IL-17 can be reversed at the GSK-3β level by PI3K/Akt signaling induced by D-resolvins. Interestingly, DEL-1 expression is reduced in aged mice, which may be related to the increased expression level of IL-17 (39, 50).

Another pro-inflammatory cytokine, TNF, can also reduce DEL-1 expression and secretion in endothelial cells by reducing C/EBPβ binding to the DEL-1 promoter, while the steroid hormone dehydroepiandrosterone (DHEA) increased DEL-1 expression and secretion in endothelial cells by activating tropomyosin receptor kinase A (TRKA) and downstream PI3K/AKT signaling to counteract the inhibitory effect of TNF and restore C/EBPβ binding to the DEL-1 promoter (51). Recently, erythromycin was reported to reverse the inhibitory effect of IL-17 on DEL-1 expression by binding to growth hormone secretagogue receptor (GHSR) and activating JAK2/MAPK p38 signaling (52). Furthermore, another independent research group found that overexpression of the p53 response element enhanced the transcriptional activity of EDIL3 (53). Primary endothelial cells isolated from p53 knockdown mice showed decreased DEL-1 mRNA expression (53). In melanoma cells, inhibition of p38/MK2 signaling reduced DEL-1 expression, suggesting that DEL-1 may be a downstream target of MK2 (54). In conclusion, the regulatory mechanism of DEL-1 expression is still unclear and needs to be further explored.

### Structure and function

**Figure 2:**
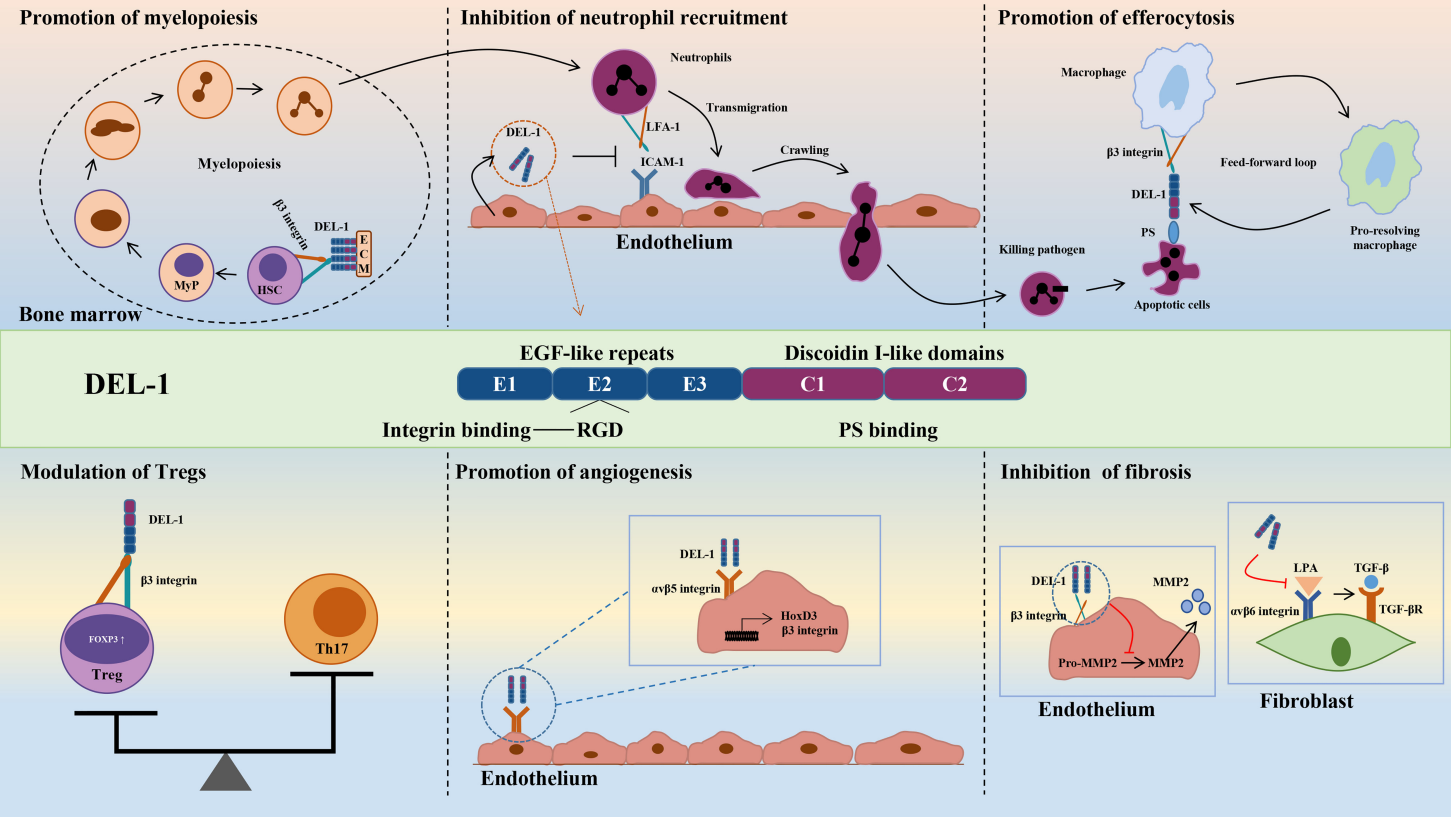
Structure and biological roles of DEL-1. The figure shows the multidomain structure of DEL-1 as well as six major regulatory activities of this protein, namely, promoting myelopoiesis, inhibiting neutrophil recruitment, promoting efferocytosis, modulating Tregs, promoting angiogenesis and inhibiting fibrosis. DEL-1, developmental endothelial locus-1; ECM, extracellular matrix; HSC, hematopoietic stem cell; MyP, myeloid progenitors; ICAM-1, intercellular adhesion molecule-1; LFA-1, lymphocyte function-associated antigen-1; PS, phosphatidyl serine; Th17, T helper 17 cell; Treg, T regulatory cell; FOXP3, forkhead box P3; HoxD3, homeobox D3; MMP2, matrix metallopeptidase 2; LPA, latency-associated peptide; TGF-β, transforming growth factor-β.

DEL-1 comprises three N-terminal EGF-like repeats (E1, E2 and E3) and two C-terminal discoidin I-like domains (C1 and C2) (44, 55). The RGD (Arg–Gly–Asp) motif in the second EGF-like repeat (E2) allows DEL-1 to interact with different integrins, including the β2 (e.g., αLβ2 and αMβ2) and β3 (e.g., αVβ3) integrins (44, 56, 57). The discoidin I-like domain and glycosaminoglycan mediate the interaction of DEL-1 with phosphatidylserine (PS) (40, 58). These interactions in turn confer important functions of DEL-1 in regulating immunity that have a major impact on the initiation and resolution of inflammation, suggesting that DEL-1 may be a promising therapeutic target (39). Specifically, the interaction of DEL-1 with αLβ2 or αMβ2 blocks the binding of the latter to its endothelial counterreceptor intercellular adhesion molecule-1 (ICAM-1), thereby inhibiting leukocyte adhesion and recruitment to sites of inflammation (46, 59). With its anti-inflammatory properties, DEL-1 can prevent a variety of inflammation-related conditions, such as multiple sclerosis and lung inflammation (45–48, 60, 61). DEL-1 can capture platelet microparticles by linking with PS and promote endothelial cell clearance of microparticles in an αVβ3 integrin-dependent manner (62). In addition, DEL-1 can act as a bridging molecule to bind PS on apoptotic cells and αVβ3 integrin on macrophages at both ends, mediating the burial of apoptotic cells and promoting inflammation resolution (40, 63). Collectively, DEL-1 exerts anti-inflammatory effects by inhibiting neutrophil recruitment and migration, promoting inflammatory resolution by accelerating macrophage reprogramming, and regulating myelopoiesis (**Figure 2**). These functions are discussed in detail in the review by Hajishengallis et al. (39, 64). Experiments with various deletion mutants of DEL-1 showed that fragments containing the C-terminus of C1 with a lectin-like structure were deposited directly in the ECM (58). The deposition efficiency varied according to the presence of other domains in DEL-1. The fragment containing E3 and C1 had the strongest deposition activity, while the fragment containing C2 was highly homologous to C1 and had low deposition activity (58). These data suggest that the discoidin domain of the DEL-1 protein contributes to its deposition and function in the extracellular matrix.

Genetic knockout or overexpression of DEL-1 in mice is an important tool in studying the function of DEL-1. EDIL3-/- mice have a specific phenotype characterized by increased development of spontaneous periodontitis (45). DEL-1 deficiency promoted neutrophil infiltration and inflammatory bone loss in mice with periodontitis (45). In experimental allergic encephalomyelitis (EAE), DEL-1 deficiency increased immune cell infiltration and inflammatory responses in the central nervous system, leading to increased disease severity (47). DEL-1-deficient mice exhibit increased neutrophil infiltration and inflammatory responses during lung inflammation (46). In postoperative peritoneal adhesion (PPA) mice, EDIL3-/- mice had a higher incidence of PPA and an increased inflammatory response, resulting in more severe PPA (65). Myelopoiesis in EDIL3-/- mice was suppressed in hematopoietic stem cells (HSCs) (66). The position of DEL-1 expression critically determines its regulatory function. In the future, the application of different transgenic mice with tissue- or cell-specific knockout or overexpression of DEL-1 may better help us to study its function.

## DEL-1 in CVDs

### Atherosclerosis

As a lipid-driven chronic inflammatory disease that underlies various CVDs, such as ischemic heart disease (IHD) (67–69), AS is caused by the accumulation and oxidative modification of low-density lipoprotein (LDL) in the arterial intima (70). As the tissue microenvironment changes, endothelial cells release chemokines and adhesion molecules, which promote the recruitment and migration of monocytes on the endothelium; monocytes subsequently differentiate into macrophages to phagocytose oxidized low-density lipoprotein (oxLDL), while the excessive accumulation of oxLDL eventually leads to the transformation of macrophages into foam cells and initiates the secretion of inflammatory cytokines to promote the development of AS plaques; moreover, smooth muscle cells migrate to the subendothelial space to form fibrous caps and stabilize the plaques. Finn et al. found that the serum level of DEL-1 in patients with coronary heart disease (3.9 ± 0.2 pg/mg total protein) was significantly higher than that in healthy subjects (2.9 ± 0.1 pg/mg total protein) (71). However, there is still a lack of clinical evidence to prove that DEL-1 is related to the occurrence and development of AS.

**Figure 3:**
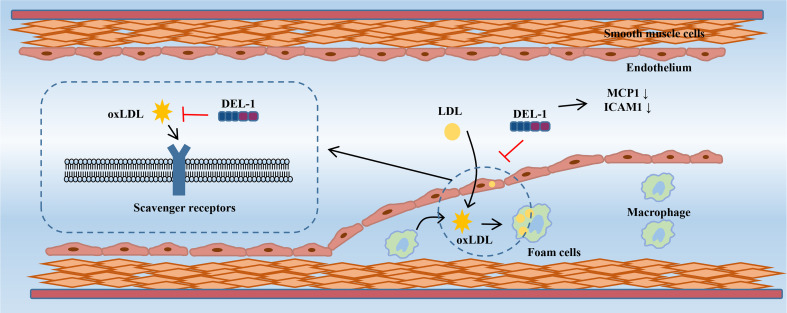
DEL-1 binds directly to oxLDL to block its binding to scavenger receptors and exert significant anti-atherogenic effects. In addition, DEL-1 reduced the expression of MCP1 and ICAM1 in the endothelial cell. DEL-1, developmental endothelial locus-1; oxLDL, oxidized low-density lipoprotein; MCP1, monocyte chemotactic protein-1; ICAM1, intercellular adhesion molecule-1.

*In vitro* evidence showed that DEL-1 can not only directly bind to oxLDL but also inhibit the uptake of oxLDL in cells transfected with multiple scavenger receptor genes in a dose-dependent manner, such as lectin-like oxidized low-density lipoprotein receptor-1 (LOX-1), scavenger receptor A (SR-A), scavenger receptor class B type I (SR-BI), and the cluster of differentiation 36 (CD36) (72). DEL-1 inhibited the uptake of oxLDL by human coronary artery endothelial cells (HCAECs) and macrophages. Furthermore, the oxLDL-induced increase in monocyte chemotactic protein-1 (MCP-1) and intercellular adhesion molecule-1 (ICAM−1) expression in HCAECs was significantly inhibited by DEL-1, which has the potential to alleviate monocyte adhesion. OxLDL-induced endothelin-1 secretion in HCAECs was also significantly inhibited by DEL-1 (72). Therefore, Del-1 not only inhibited the binding of oxLDL to the receptors but also inhibited the cellular response to oxLDL (**Figure 3**).

In a mouse model of AS, DEL-1 overexpression inhibited the receptor-binding activity of a modified LDL in serum, reduced the expression of adhesion molecules MCP-1 and ICAM-1 in the aorta, and reduced the oil red O-positive atherosclerotic area at the aortic roots (72). These results suggest that DEL-1 overexpression inhibits the occurrence of AS. However, in contrast to the above results, Subramanian et al. constructed an AS model by partially ligating the left carotid artery in ApoE^−/−^ mice and found that endothelial cell-specific overexpression of DEL-1 had no significant effect on the development and cellular composition of AS plaques (73). These researchers fed ApoE^−/−^ mice a high-fat diet for 4 or 12 weeks to study early or late lesions and found that endothelial cell-specific overexpression of DEL-1 did not affect early or late stages of AS and did not prevent AS (73). The apparent discrepancy between the results of this study and those of Kakino et al. may be due to the following: 1. The transgenic mice in Kakino et al.’s study overexpressed DEL-1 in all cell types. In addition to the mechanism mediated by endothelial cell-derived DEL-1, other mechanisms may also play a role, such as macrophages. 2. Differences in experimental methods between the two studies may also lead to conflicting results, such as differences in the background of ApoE^−/−^ mice, differences in high fat diets, differences in modeling methods, and so on. In the future, transgenic mice with macrophage-specific expression may help to further elucidate the role of DEL-1 in AS.

Intercellular signaling plays a key role in AS formation, affecting the occurrence and progression of CHD, and circulating microRNAs (miRNAs) may be involved in this process (74). There were clear differences in circulating miRNA transport between CHD patients and healthy subjects, especially the reduction in miRNA enrichment in microparticles (MPs) (71, 75). Furthermore, MPs from CHD patients were less efficient at transferring miRNAs to cultured HUVECs, suggesting that MP uptake is impaired in the disease state. DEL-1 can mediate the uptake of MPs by endothelial cells by binding to PS on the external surface of MPs (62, 63). Although circulating levels of DEL-1 are increased in CHD patients, these patients have less DEL-1 binding to MPs (71). Therefore, Finn et al. suggested that DEL-1 binding to MPs was impaired in the serum of individuals with CHD, thereby altering circulating miRNA transport and affecting CHD initiation and progression. In the future, in addition to regulating the expression of DEL-1, regulating the function of DEL-1 may be an important aspect in the treatment of AS.

### Hypertension

Hypertension refers to a clinical syndrome characterized by increased systemic arterial blood pressure (systolic and/or diastolic blood pressure), which may be accompanied by functional or organic damage to organs such as the heart, brain, and kidneys (76). Hypertension is the most common chronic disease and the main risk factor for cardiovascular and cerebrovascular diseases (77). Although the pathophysiological mechanisms of hypertension are not fully understood, strong evidence suggests that immune hyperactivation and chronic inflammatory responses play a crucial direct role in the development of hypertension (78). Our team’s previous clinical and animal studies also proved that immune microenvironment disturbances are closely related to hypertension (79–83). Activated T lymphocytes and proinflammatory cytokines such as IL-17 are involved in the occurrence and development of angiotensin II (ANGII) and deoxycorticosterone acetate-salt (DOCA-salt)-induced hypertension (84–89). Gene knockout or neutralization with antibodies against IL-17 limited the progression of hypertension (86, 88, 90). DEL-1 can inhibit inflammation through various anti-inflammatory effects to alleviate IL-17-mediated conditions, such as inflammatory bone loss and multiple sclerosis, suggesting that DEL-1 may be a potential target for the treatment of hypertension (45, 48). Furthermore, DEL-1 promoted vascular smooth muscle cell (VSMC) adhesion, migration and proliferation in a dose-dependent manner, and this process was mediated through αVβ_3_ integrin (91). These data suggested that DEL-1 has a paracrine role in vascular remodeling.

Recently, Failer et al. found that endothelial DEL-1-overexpressing mice had less adventitial collagen, lower medial thickness, and more elastin, suggesting that DEL-1 overexpression prevents ANGII-induced aortic remodeling (41). DEL-1 overexpression also prevented the progression of ANGII-induced hypertension, endothelial dysfunction and aortic fibrosis. DEL-1 overexpression alleviated the infiltration of CD45 leukocytes, TCR-β T cells and CD45IL-17 leukocytes in the aorta after ANGII infusion. Moreover, DEL-1 overexpression inhibited the expression of proinflammatory cytokines induced by ANGII and increased the expression level of the anti-inflammatory cytokine IL-10. In addition to inflammation, DEL-1 overexpression inhibits the activity of matrix metallopeptidase 2 (MMP2) in the aorta, whose increase critically contributes to aortic remodeling in hypertension (92, 93).

Failer et al. next investigated the preventive and therapeutic effects of recombinant DEL-1-FC on ANGII-induced hypertension. Intervention with recombinant DEL-1-FC administered before or after hypertension prevented or eliminated ANGII-induced aortic remodeling, hypertension, arterial stiffness, and inflammation (41). Recombinant DEL-1-FC also inhibited the activity of MMP2 in the aorta while promoting the infiltration of anti-inflammatory Tregs. Failer et al. also found that the mutation of the RGE part of DEL-1 abolished the protective effect of DEL-1-FC, suggesting that RGE is involved in the pathophysiological process of DEL-1 inhibiting the occurrence and development of hypertension. In a DOCA salt-induced hypertension model, recombinant DEL-1 treatment similarly attenuated aortic remodeling, hypertension, and inflammatory progression and promoted Treg infiltration (41).

**Figure 4:**
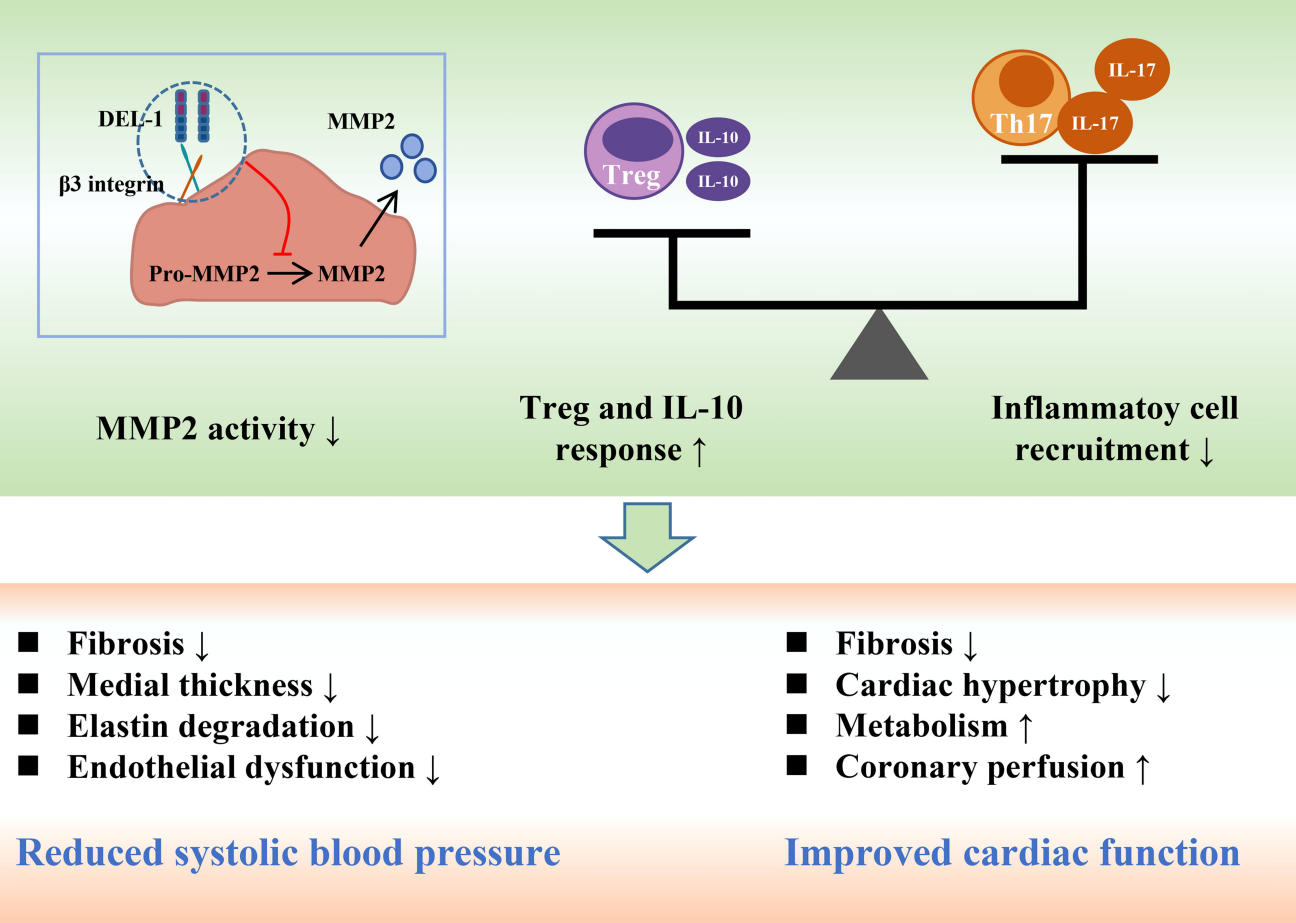
DEL-1 reduces blood pressure and maintains cardiac function by inhibiting MMP2 activity, reducing inflammatory cell infiltration and enhancing Treg and IL-10 responses. DEL-1, developmental endothelial locus-1; MMP2, matrix metallopeptidase 2; Th17, T helper 17 cell; Treg, T regulatory cell; IL, interleukin.

A series of *in vitro* experiments further demonstrated that DEL-1 overexpression and recombinant DEL-1 treatment inhibited ANGII-induced activation of MMP2 in human and mouse vascular tissues, which was αVβ3 integrin-dependent (41, 94). Correspondingly, RGE mediates the binding of αVβ3 integrin to DEL-1, which may explain the abolition of the protective effect of DEL-1 by RGE mutation (41, 56). In conclusion, the findings of Failer et al. fully demonstrate the protective role of DEL-1 in the occurrence and development of hypertension and suggest this molecule may become a potential drug for the treatment of hypertension in the future (**Figure 4**).

### Cardiac remodeling

Cardiac remodeling is an independent risk factor for heart failure, arrhythmias, and sudden death and is a key determinant of the clinical course and long-term prognosis of patients with CVDs (95). Pathological cardiac remodeling is characterized by cardiomyocyte hypertrophy and interstitial fibrosis under various cardiac stresses, such as hypertension and MI, resulting in increased myocardial stiffness and impaired cardiac contractility (96, 97). Cardiac remodeling is associated with fibrosis, capillary sparseness, increased production of proinflammatory cytokines, cellular dysfunction (impaired signaling, inhibition of autophagy, and abnormal cardiomyocyte/noncardiomyocyte interactions), and adverse epigenetic alterations (95). Our previous studies further shed light on the pathogenesis of cardiac remodeling, suggesting that inhibition of cardiac remodeling by pharmacological or genetic approaches significantly improves cardiac dysfunction and survival (21, 98–101).

In mice, fibroblasts constituted 27% of all cardiac cells, contributing to the maintenance of homeostasis under physiological conditions and regulating tissue remodeling in response to stress (95, 102, 103). Pathological fibrosis results from abnormal regulation of extracellular matrix (ECM) production in tissues or organs, including collagen (97). Compared with that in normal lung tissue, the expression level of DEL-1 was decreased in lung fibrous tissue, suggesting that DEL-1 may be associated with pulmonary fibrosis (60). DEL-1 deficiency promoted collagen synthesis and secretion by regulating transforming growth factor (TGF-β), thereby aggravating bleomycin-induced pulmonary fibrosis (60, 104). Yan et al. found that DEL-1-deficient mice had a higher incidence of postoperative peritoneal adhesions, accompanied by enhanced collagen production (65). In contrast, DEL-1 supplementation reduced the incidence and severity of postoperative peritoneal adhesions. *In vitro* studies have demonstrated that DEL-1 inhibits TGF-β activation in 293T cells and RAW264.7 mouse macrophages by binding to αVβ6 integrin (104). These data suggest that DEL-1 plays an important role in the initiation and progression of tissue fibrosis.

The immune system and inflammatory response mediate pathological cardiac remodeling (97). Immunomodulation may be an important strategy to alleviate cardiac remodeling. Failer et al. found that endothelial DEL-1 overexpression or recombinant DEL-1 treatment inhibited AGNII or DOCA salt-induced inflammation and MMP2 activation in the heart, thereby reducing cardiac hypertrophy, fibrosis, and dysfunction (41). However, cardiac remodeling in this study belongs to target organ damage caused by hypertension, and the regulation of DEL-1 on blood pressure may indirectly affect cardiac remodeling. Therefore, this study may have certain limitations. Future studies on cardiac remodeling may help us further understand the function of DEL-1.

### Ischemic heart disease

**Figure 5:**
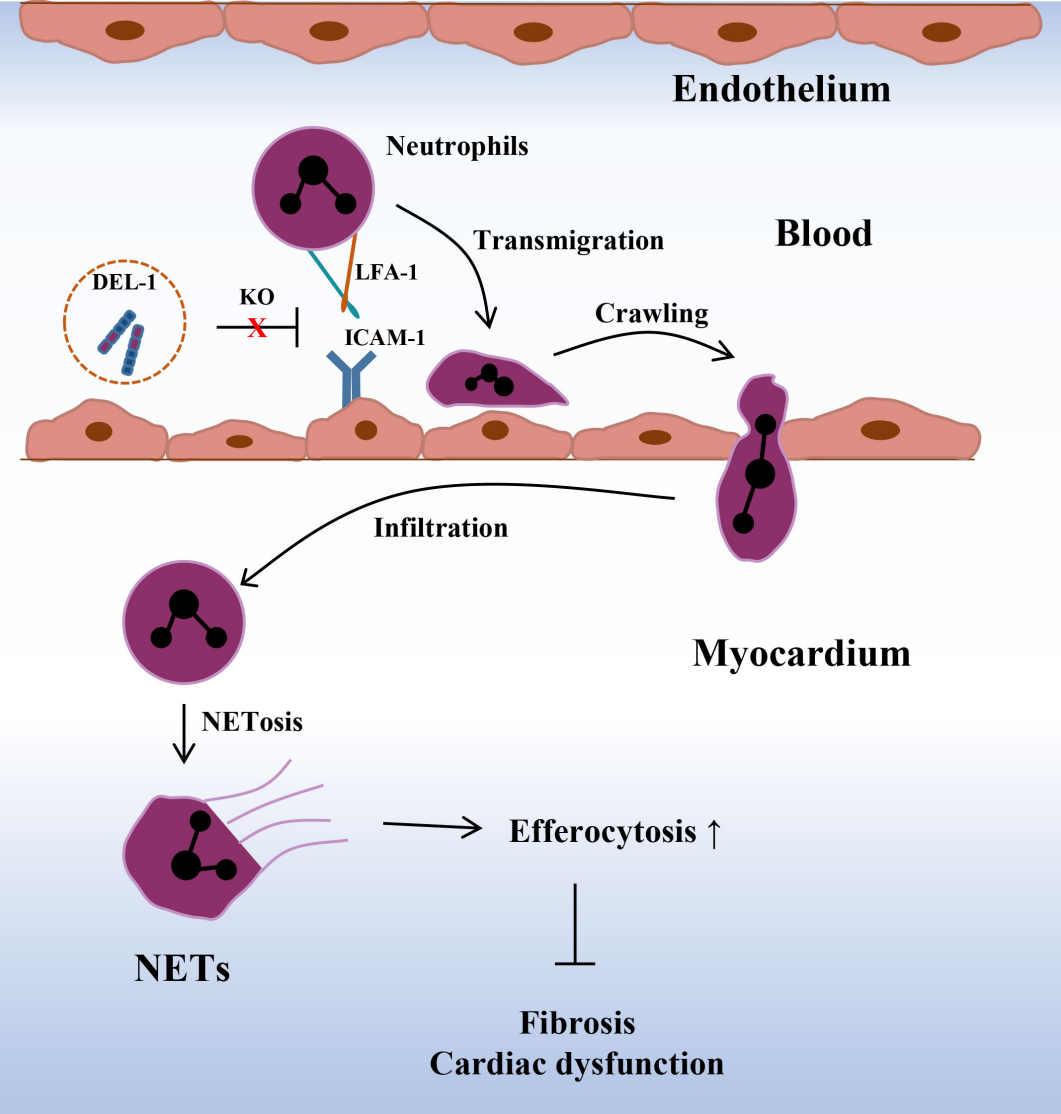
Deletion of DEL-1 promotes neutrophil infiltration and formation of NETs, thereby promoting macrophage efferocytosis and alleviating cardiac fibrosis and cardiac dysfunction after MI. DEL-1, developmental endothelial locus-1; NETs, neutrophil extracellular trap; MI, myocardial infarction.

Ischemic heart disease (IHD), mainly caused by coronary atherosclerosis and its complications, can induce congestive HF and life-threatening arrhythmias and is the leading cause of death worldwide (105, 106). Acute myocardial infarction (AMI) is the most serious IHD with the highest mortality rate (106). In a pig model of cardiac ischemia induced by left circumflex artery ligation, DEL-1 treatment improved cardiac function (107). Wei et al. found that DEL-1 levels were decreased in severe AMI patients, which is consistent with the finding thar WT mice with MI showed low levels of cardiac DEL-1 (42). Compared with WT mice, DEL-1-/- mice showed significantly improved cardiac function and alleviated cardiac remodeling post-MI. Mechanistically, the protective effect of DEL-1 deficiency in MI was associated with enhanced neutrophil recruitment and expansion of proinflammatory monocyte-derived macrophages (42). Injection of a neutrophil-specific C-X-C motif chemokine receptor 2 (CXCR2) antagonist impaired macrophage polarization, increased cellular debris and exacerbated adverse cardiac remodeling, thereby abrogating the protective effect of DEL-1 deficiency. Inhibition of neutrophil extracellular trap (NET) formation by treatment with a neutrophil elastase inhibitor or DNase I abrogated differences in macrophage polarization and cardiac function between WT and DEL-1-/- mice after MI. Collectively, these data suggest that DEL-1 is a key regulator of neutrophil recruitment and macrophage polarization during cardiac remodeling after MI (**Figure 5**).

Increasing evidence has shown that healing of MI involves a series of delicately regulated inflammatory responses (108). Following MI, injured cardiomyocytes release damage-associated molecular patterns (DAMPs), cytokines, and chemokines, leading to substantial recruitment of neutrophils and monocytes/macrophages to the myocardium (109, 110). These neutrophils and monocytes contribute to the removal of debris and dead cells, as well as the activation of repair pathways. Furthermore, recruited monocytes give rise to proinflammatory or repairing macrophages. Proinflammatory macrophages produce cytokines, release MMPs to promote extracellular matrix destruction, and clear cellular debris, while repairing macrophages promotes fibroblast-to-myofibroblast transformation and enhances collagen deposition, leading to the formation of crosslinked collagen (111). A scar is formed to protect the left ventricle (LV) from rupture of the heart. Wei et al. confirmed the integral role of inflammation in the healing process (42). However, excessive inflammation may exacerbate MI-induced myocardial injury (109, 111). DEL-1 has anti-inflammatory and proresolving effects, and the lack of DEL-1 may inhibit inflammatory resolution, leading to an excessive inflammatory response and exacerbating tissue damage (40, 60, 112, 113). Therefore, the extent of the increased inflammation caused by DEL-1 deficiency in Wei et al.’s study requires further scrutiny.

The study by Wei et al. is the only report of amelioration of DEL-1 deficiency (42). In previous reports, inhibition of neutrophil recruitment improved cardiac dysfunction and cardiac remodeling after MI (114–116). Inhibition of neutrophils by DEL-1 also exerted protective effects in other diseases, which seems to contradict the study by Wei et al. (46, 48, 117). Multiple actions of DEL-1, such as anti- and proinflammatory resolution (39), coronary vasodilation (41), inhibition of MMP2 activity (41) and promotion of angiogenesis (118, 119), may protect the heart from MI-induced injury. The study by Wei et al. has certain limitations, such as the lack of cell-specific gene-edited mice and the lack of analysis of preventive or therapeutic effects of recombinant DEL-1 (42). The future use of endothelial or macrophage-specific DEL-1 transgenic mice and recombinant DEL-1 may help us further understand the role and mechanism of DEL-1 in IHD.

### Other cardiovascular diseases

DEL-1 was found to regulate vascular morphogenesis or remodeling in embryonic development as early as 1998 when it was first cloned and characterized (44). DEL-1 provides a unique autocrine angiogenic pathway for the embryonic endothelium, which is mediated in part by integrin α_v_β_3_ (120). DEL-1 mediates VSMC adhesion, migration and proliferation through interaction with integrin α_v_β_3_, which may regulate vascular wall development and remodeling (91). Aoka et al. found that DEL-1 accelerates tumor growth by promoting enhanced angiogenesis (121). Expression of endogenous DEL-1 protein is increased in ischemic hindlimbs (122). DEL-1 binding to α_v_β_5_ upregulated the expression of the transcription factor Hox D3 and integrin α_v_β_3_, thereby promoting angiogenesis and functional recovery in a hindlimb ischemia model (57). Exogenous intramuscular administration of DEL-1 significantly enhanced angiogenesis in ischemic hindlimbs in mice, suggesting that DEL-1 may be a novel therapeutic agent for ischemic patients (122). A clinical study compared VLTS-589 (a plasmid encoding Del-1 conjugated to poloxamer 188) with the poloxamer 188 control in the treatment of intermittent claudication in patients with moderate to severe peripheral arterial disease (123). Intramuscular delivery of a plasmid expressing DEL-1 and the control significantly improved baseline exercise capacity at 30, 90, and 180 days, but there was no difference in outcome measures between the two groups. DEL-1-mediated angiogenesis has also been reported in many other diseases, such as ischemia models, lung adenocarcinoma, retinopathy, squamous cell carcinoma, and psoriasis (119, 124–129). Taken together, these data suggest that DEL-1-regulated angiogenesis may be a target for many diseases, but its clinical value requires further clinical trials.

Similar to MI, strokes are also caused by vascular or microvascular diseases that disrupt the blood supply to the brain, leading to brain dysfunction (130). The number of new vessels generated in ischemic brain tissue is associated with decreased morbidity and longer survival in stroke patients, suggesting that restoration of cerebral microvascular circulation is important for functional recovery after ischemic attacks (131). DEL-1 expression was increased in the ischemic cortical peri-infarct area after ischemic stroke (118). DEL-1 gene transfer induced cerebral angiogenesis and may be a novel and effective method for stimulating cerebral angiogenesis after stroke (118). Electroconvulsive seizures (ECSs) have been shown to treat major depression by modulating neurotrophy and angiogenesis (132, 133). Newton et al. found that ECS treatment increased DEL-1 expression in brain tissue and promoted angiogenesis in the adult rat hippocampus (134). In conclusion, DEL-1-mediated angiogenesis may be one of the targets for the treatment of cerebrovascular diseases.

## DEL-1 in metabolic diseases

The prevalence of metabolic diseases, including diabetes, is increasing, while the westernization of dietary habits has led to an increase in obesity (135). Obesity-related chronic low-grade inflammation has been reported to cause insulin resistance in muscle, liver, and adipose tissue (136). Insulin resistance refers to the decrease in the efficiency of insulin to promote glucose uptake and utilization for various reasons, and the compensatory secretion of excessive insulin produces hyperinsulinemia to maintain the stability of serum glucose levels (137). Insulin resistance predisposes patients to metabolic syndrome and type 2 diabetes. DEL-1 ameliorates palmitate-induced endoplasmic reticulum (ER) stress and insulin resistance in the mouse skeletal muscle cell line C2C12 *via* SIRT1/SERCA2-related signaling (43). *In vivo* experiments showed that DEL-1 administration increased the expression of SIRT1 and SERCA2, thereby ameliorating insulin resistance in skeletal muscle of high fat diet (HFD)-fed mice and improving HFD-impaired glucose tolerance and insulin sensitivity (43). These results suggest that DEL-1 may be a novel therapeutic target for the management of insulin resistance and type 2 diabetes.

Regular exercise is the treatment of choice for obesity and obesity-mediated metabolic disorders such as insulin resistance, type 2 diabetes, atherosclerosis, and hypertension (138). Compared with that in healthy subjects, DEL-1 mRNA expression was decreased in the muscle of obese and diabetic patients (139). Exercise increases DEL-1 mRNA expression levels in obese/diabetic patients in a time-dependent manner (139). DEL-1 secreted by exercising skeletal muscle can affect various tissues through the bloodstream, including adipose tissue (140). *In vitro* experiments showed that DEL-1 attenuated palmitate-induced inflammation and insulin signaling impairment in adipocytes by regulating AMPK/HO-1 signaling (139). In addition, DEL-1 treatment promoted AMPK phosphorylation and enhanced adipocyte thermogenesis but did not affect intracellular lipid accumulation (139).

In another endometrial cancer (EC) cohort study, Cobb et al. found an association between patient BMI and increased DEL-1 expression in cancer tissue (141). Furthermore, HFD increased the expression of DEL-1 in tumors compared with a low-fat diet in EC model mice (141). These data suggest that DEL-1 may serve as a novel obesity-driving target that should be further explored in future research work.

Thus, DEL-1-mediated anti-inflammatory and proresolving effects provide a basis for the amelioration of metabolic diseases. DEL-1 is involved in the regulation of obesity and insulin resistance. However, the current relevant evidence is still insufficient, and more research is needed in the future to reveal the role of DEL-1 in metabolic diseases.

## Concluding remarks and future perspectives

DEL-1 has received considerable attention since it was first cloned and characterized as a factor promoting embryonic angiogenesis (44). DEL-1 is widely expressed in different tissues, such as the brain, lung and blood vessels, to maintain tissue homeostasis. As a secreted protein, the serum level of DEL-1 may be related to the diagnosis and prognosis of various diseases, such as MI, sepsis and osteoarthritis (42, 142, 143). As a local tissue signal, DEL-1 exerts anti-inflammatory and proresolving effects in different tissues and stages, thereby ameliorating a variety of inflammation-related diseases (39). Emerging studies over the past few years have convincingly demonstrated that DEL-1 has a therapeutic effect on a variety of CVMDs, including AS, hypertension, cardiac remodeling, and insulin resistance. This review summarizes the potential involvement of DEL-1 in cardiovascular and metabolic homeostasis, thereby defining DEL-1 as a promising biomarker and therapeutic target for CVMDs.

Despite our detailed understanding of the role of DEL-1 in various pathophysiological processes, several questions remain to be answered. We propose some solutions to these questions in this review. First, systemic overexpression rather than endothelial cell-specific overexpression of DEL-1 inhibited the occurrence and development of AS, and the mechanism remains unclear (72, 73). Other cells, such as macrophage-specific overexpression mice, may help us understand the role of DEL-1 in AS. Future basic research on the use of recombinant DEL-1 in the treatment of AS can provide a reference for its clinical application. Second, although Wei et al. found that DEL-1 treatment attenuated hypertension-induced cardiac remodeling, this protective effect may be attributable to reduced blood pressure (41). More direct evidence for the treatment of DEL-1 in cardiac remodeling is lacking. The application of other cardiac remodeling models could better reveal the therapeutic effect of DEL-1 on cardiac remodeling. *In vitro* experiments can also help us further understand the mechanism by which DEL-1 treatment improves cardiac remodeling. Third, DEL-1 deficiency ameliorated cardiac dysfunction and remodeling in MI by promoting inflammation (42). Although the data in this study are sufficient, we remain concerned about the extent of increased inflammation caused by DEL-1 deficiency, as excessive inflammation is damaging. Future treatment with DEL-1 overexpression or recombinant protein may help us to further understand the role and mechanism of DEL-1 in MI.

The protective effect of DEL-1 in CVMDs has important clinical value. There is currently only one phase II, multicenter, double-blind, placebo-controlled study of DEL-1 in the treatment of intermittent claudication, which combined a plasmid encoding DEL-1 with poloxamer 188 to form VLTS-589 and delivered this treatment intramuscularly (123). Although the outcomes of DEL-1 plasmid-treated patients did not change compared with the controls, this was an important attempt at clinical application of DEL-1. Some researchers have also used DEL-1 for tissue engineering to promote angiogenesis (119, 124). On the one hand, we can use gene therapy that promotes the expression of DEL-1 by constructing plasmids for clinical experiments, and on the other hand, we can also use nanomaterials and other technologies to deliver recombinant DEL-1 protein or plasmids to target tissues, such as the heart and brain. In addition, well-designed, large-scale, high-quality, and multicenter clinical trials are needed to evaluate the safety, toxicological profile, and clinical utility of DEL-1 in human patients with CVMDs.

Collectively, DEL-1 is a promising biomarker and therapeutic target for CVMDs.

## Author contributions

JW and MW participated in the design of the project. MZ and CL were responsible for drafting the manuscript. ZZ was responsible for the figures of this review. All authors contributed to the article and approved the submitted version.

## Funding

This work was supported by grants from the National Natural Science Foundation of China (82070436, 82100292, 82270454).

## Conflict of interest

The authors declare that the research was conducted in the absence of any commercial or financial relationships that could be construed as a potential conflict of interest.

## Publisher’s note

All claims expressed in this article are solely those of the authors and do not necessarily represent those of their affiliated organizations, or those of the publisher, the editors and the reviewers. Any product that may be evaluated in this article, or claim that may be made by its manufacturer, is not guaranteed or endorsed by the publisher.
